# Multiple Antenatal Dexamethasone Treatment Alters Brain Vessel Differentiation in Newborn Mouse Pups

**DOI:** 10.1371/journal.pone.0136221

**Published:** 2015-08-14

**Authors:** Winfried Neuhaus, Marian Schlundt, Markus Fehrholz, Alexander Ehrke, Steffen Kunzmann, Stefan Liebner, Christian P. Speer, Carola Y. Förster

**Affiliations:** 1 Department of Anaesthesia and Critical Care, University of Wuerzburg, Wuerzburg, Germany; 2 Department of Pharmaceutical Chemistry, University of Vienna, Vienna, Austria; 3 Institute of Medical Genetics, Medical University of Vienna, Vienna, Austria; 4 University Children’s Hospital Wuerzburg, Wuerzburg, Germany; 5 Institute of Neurology (Edinger Institute), Johann Wolfgang Goethe University, Frankfurt, Germany; Texas A&M University Health Science Center College of Medicine & Baylor Scott and White Health, UNITED STATES

## Abstract

Antenatal steroid treatment decreases morbidity and mortality in premature infants through the maturation of lung tissue, which enables sufficient breathing performance. However, clinical and animal studies have shown that repeated doses of glucocorticoids such as dexamethasone and betamethasone lead to long-term adverse effects on brain development. Therefore, we established a mouse model for antenatal dexamethasone treatment to investigate the effects of dexamethasone on brain vessel differentiation towards the blood-brain barrier (BBB) phenotype, focusing on molecular marker analysis. The major findings were that in total brains on postnatal day (PN) 4 triple antenatal dexamethasone treatment significantly downregulated the tight junction protein claudin-5, the endothelial marker Pecam-1/CD31, the glucocorticoid receptor, the NR1 subunit of the N-methyl-D-aspartate receptor, and Abc transporters (Abcb1a, Abcg2 Abcc4). Less pronounced effects were found after single antenatal dexamethasone treatment and in PN10 samples. Comparisons of total brain samples with isolated brain endothelial cells together with the stainings for Pecam-1/CD31 and claudin-5 led to the assumption that the morphology of brain vessels is affected by antenatal dexamethasone treatment at PN4. On the mRNA level markers for angiogenesis, the sonic hedgehog and the Wnt pathway were downregulated in PN4 samples, suggesting fundamental changes in brain vascularization and/or differentiation. In conclusion, we provided a first comprehensive molecular basis for the adverse effects of multiple antenatal dexamethasone treatment on brain vessel differentiation.

## Introduction

The use of antenatal steroids to mature fetal lungs reduces neonatal morbidity and mortality in neonates born before 34 weeks of gestation [1,2]. Clinical trials have shown that a single course of antenatal glucocorticoids (e.g., betamethasone or dexamethasone) reduces the incidence of neonatal death, respiratory distress syndrome, intraventricular hemorrhage, necrotizing enterocolitis and early sepsis, and increases the efficacy of postnatal surfactant therapy compared with placebo [1]. However, the use of repeated courses of antenatal glucocorticoids is more controversial [2,3]. There is concern that a prolonged induction of tissue differentiation may result in inappropriate patterns of fetal organ growth that have consequences for later health [4]. Animal studies have shown that exposure to repeated or high-dose antenatal glucocorticoids is associated with reduced fetal growth and long-term adverse effects on brain development, neuroendocrine function, blood pressure, and glucose homeostasis [5]. The negative consequences of high dose steroid administration aiming at accelerated lung maturity may be a loss of brain cells and increased neurodevelopmental disability [4]. Excessive dexamethasone treatment could lead to white matter lesions with distinct demyelination in infants. Oligodendrocytes play a pivotal role in myelin formation, which is dependent on the maturation status of oligodendrocytes and their progenitors. Excessive dexamethasone treatment may influence this maturation process [6]. Recent studies have highlighted the importance of the crosstalk between the brain endothelium and oligodendrocytes. It has been proposed that a disruption of the trophic coupling between brain endothelial cells and oligodendrocytes could lead to white matter dysfunction [7]. This hypothesis led us to investigate the effects of antenatal maternal dexamethasone treatment on the development of the blood-brain barrier (BBB).

The BBB plays a crucial role in central nervous system (CNS) homeostasis, and disturbances of BBB development may lead to adverse CNS development. The BBB consists *per definitionem* of brain capillary endothelial cells, which are regulated by their microenvironment (e.g., astrocytes, pericytes, and shear stress applied by blood flow) [8,9]. Recent studies have revealed that the response of brain capillary endothelial cells to several stimuli is dependent on their developmental status [10]. Single maternal treatments with glucocorticoids, such as dexamethasone, led to the increased expression of tight junction proteins, such as claudin-5, in fetal sheep brains [11,12], but multiple dexamethasone treatment did not significantly regulate claudin-5. Specific tight junction proteins seal the intercellular gaps between brain capillary endothelial cells, which significantly tightens the paracellular barrier. A decrease or loss of these tight junction proteins could increase permeability. This relationship between tight junction protein expression and BBB permeability was also observed in dexamethasone-treated fetal sheep [12]. However, comprehensive data on changes of the BBB induced by maternal antenatally applied glucocorticoids, are absent. Consequently, we established a mouse model to investigate the effects of single and multiple maternal antenatal dexamethasone treatments on key BBB molecules during development. The dose of dexamethasone (0.1 mg/kg body weight) and injection days (single: E16; multiple: E15, E16, E17) were chosen according to clinical relevance. Same doses were also used in previous studies with pregnant mice [13]. Brains of postnatal day four (PN4) and 10 (PN10) mouse pups were investigated since PN4 could be used as a model for the developmental status of pre-term and PN10 as a model for term human newborns, respectively [14]. Results showed that multiple antenatal treatment with dexamethasone reduced the expression of some BBB markers such as tight junction proteins, receptor and transporter proteins on PN4. In addition, in line with altered vessel morphology shown by immunofluoresence microscopy data revealed changes of key molecules of angiogenesis as well as genes of the sonic hedgehog (Shh) and the wingless int (Wnt) pathways on PN4.

## Materials and Methods

### Animals

Timely mated pregnant C57Bl/6JRccHsd mice were purchased from Harlan (Harlan Laboratories GmbH, An Venray, The Netherlands) and delivered at embryonic day 8 (E8). The day after mating night was defined as day E0. Animals were held in scantainer ventilated cabinets (Scanbur-BK) with exercise wheels and plastic houses at the animal facility of the Department of Experimental Surgery. The animals were exposed to a 12-hour dark/light interval and ingested H_2_O and feed (Altromin, Spezialfutter GmbH & Co.Kg, Lage, Germany) *ad libitum*. Animal care before and during experiments was strictly performed in compliance with institutional guidelines of the University Hospital Würzburg, Germany. The Animal Ethics Committee of the Regierung von Unterfranken approved all experiments (protocol number 55.2–2531.01-60/10), all efforts were made to minimize suffering.

### Experimental protocols

For single treatments, pregnant mice were injected intraperitoneal (i.p.) on day E16. For triple treatments, pregnant mice were injected i.p. on days E15, E16 and E17 with either 0.1 mg/kg body weight dexamethasone (DEX) or 0.9% NaCl (+0.1% EtOH) as a control solution using Omnican-40 syringes (0.3 x 12 mm, Ref. 9161627, B.Braun, Melsungen, Germany) (S1 Fig). A fresh 15 mg/mL ethanolic DEX stock solution was diluted with 0.9% NaCl (1299.99.910541–1, DIACO, Trieste, Italy) to a 0.015 mg/mL DEX injection solution, which was sterile-filtered, aliquoted and stored in sterile caps at -20°C until injection. A 0.9% NaCl control solution was supplemented with sterile ethanol to achieve the same ethanol concentration (0.1%) as the DEX injection solution. Each pregnant mouse was held in its own cage after receiving i.p. injections. At postnatal (PN) days 4 and 10, pups were sacrificed either by single decapitation with scissors (PN4) or cervical dislocation followed by decapitation with scissors (PN10). Brains were removed and frozen directly in a pre-cooled isopentane solution in dry ice. After 10 minutes, brains were wrapped with labeled aluminum foil and stored at -80°C until further analysis. In total 40 pregnant mothers were injected, and 259 pups were born from which PN4 and PN10 brains were collected and frozen. For method development and data acquisition for the presented results brains from 173 pups were analyzed. For the presented results following animal numbers per experimental group were used: 1x NaCl PN4 (5 pregnant mothers, qPCR: 10 pups, western blotting: 6 pups), 1x DEX PN4 (4 pregnant mothers, qPCR: 12 pups, western blotting: 6 pups), 3x NaCl PN4 (8 pregnant mothers, qPCR: 13 pups, western blotting: 6 pups, immunofluorescence microscopy: 6 pups), 3x DEX PN4 (9 pregnant mothers, qPCR: 13 pups, western blotting: 6 pups, immunofluorescence microscopy: 6 pups), 1x NaCl PN10 (3 pregnant mothers, qPCR: 6 pups, western blotting: 6 pups), 1x DEX PN10 (3 pregnant mothers, qPCR: 6 pups, western blotting: 6 pups), 3x NaCl PN10 (3 pregnant mothers, qPCR: 6 pups, western blotting: 6), 3x DEX PN10 (3 pregnant mothers, qPCR: 6 pups, western blotting: 6 pups). Sample size calculation was according to the experimental approvement of the Animal Ethics Committee of the Regierung von Unterfranken (protocol number 55.2–2531.01-60/10). No adverse effects due to the treatments on animal welfare were observed.

Average litter size was six pups. Average weight of injected pregnant mice was 25–35 grams, average weight of PN4 pups was 2.70 ± 0.45 grams (mean ± SD), and average weight of PN10 pups was 5.88 ± 0.47 grams (mean ± SD).

### Isolation of brain endothelial cells

Pools of two or three PN4 brains or single PN10 brains were weighed, cooled in ice-cold HBSS and diced using a scalpel in a Petri dish on ice into maximum 1-mm pieces. Brain tissues were suspended in 1 mL ice-cold HBSS in an Eppendorf vial before the addition of 10 μl of a 0.1 g/mL collagenase/dispase solution (10269638001, Roche, Mannheim, Germany). After a 15-minutes enzymatic digestion at 37°C, suspensions were drawn through needles with decreasing diameters (21G, REF304432, 23G, REF300800, 25G, REF300400, Becton Dickinson, Heidelberg, Germany), followed by a second and a third 15-minutes incubation period, with subsequent homogenization through the needles mentioned above. Brain homogenates were centrifuged at 1000×*g* for 7 minutes at 4°C, and the pellets were washed with 1 mL ice-cold HBSS per vial three times (1000×*g*, 4°C, 7 minutes). The pellets were resuspended in 1 mL ice-cold HBSS, and one 200-μl sample was taken for qPCR analysis of the total brain before brain endothelial cell isolation. The residual 800 μl of brain homogenate was recentrifuged (1000×*g*, 4°C, 7 minutes), and the pellets were resuspended in 500 μl of Pecam-1 antibody (ab32457-100, GR18226-1, Abcam plc, Cambridge, United Kingdom)-bound dynabeads (122.03D, M280, goat, Invitrogen, Darmstadt, Germany), which were prepared according to the manufacturer’s instructions. Brain homogenate-dynabeads mixtures were incubated at 4°C with gentle shaking for 30 minutes. Cells binding to the dynabeads were isolated using a DynaMagTM Spin magnet (Invitrogen) and washed 5 times with 1 mL ice-cold HBSS to remove residual, non-binding cells. Total brain samples and brain endothelial fractions were directly lysed in 350 μL RNA-lysis buffer RA1 supplemented with 1% β-mercaptoethanol and stored at -80°C before RNA isolation, cDNA production and qPCR analysis.

### Quantitative polymerase chain reaction (qPCR)

Total RNA was isolated using the Nucleospin-RNAII Kit according to the manufacturer’s instructions. RNA concentrations were determined using a Nanodrop ND 2000 spectrophotometer (Fisher Scientific, Schwerte, Germany) at 260/280 nm. The same amounts of RNA from compared samples were reverse transcribed to 20 μL cDNA using a high-capacity cDNA-kit from Applied Biosystems (Life Technologies GmbH, Darmstadt, Germany) according to the manufacturer’s instructions. qPCR analysis was performed using FAM-labeled Taqman probes (S1 Table). The total volume per well for qPCR (25 μL) consisted of 12.5 μL absolute QPCR ROX mix (AB-1138, Applied Biosystems, Darmstadt, Germany), 1.25 μL 20x Taqman probe, 6.25 μL nuclease-free H_2_O and 5 μL of 1:10 diluted cDNA. The following qPCR time program was used: 15 minutes at 95°C, 50 cycles with 15 seconds at 95°C and 1 minute at 60°C. qPCR analyses were conducted using a 7300 Real-Time PCR System (Applied Biosystems). Each sample was analyzed in triplicate. The mRNA abundances relative to the endogenous control were calculated using the ddCt method and the following formula: 2^(Ct of 18 S rRNA-Ct of gene of interest), where Ct is the threshold cycle value. Method development revealed that all steps (i.e., organ sampling, storage, homogenization, endothelial cell isolation, cell lysis, RNA isolation, and cDNA production) had to be conducted at the same time for samples that were compared with one another for proper data analysis. β-actin, 18srRNA and glyceraldehyde 3-dehydrogenase (GAPDH) were tested as endogenous controls. The results showed a significant depletion of 18SrRNA and β-actin after brain endothelial cell isolation compared with total brains, which indicated varying amounts of these markers in different cell types (S2 Fig). By contrast, GAPDH expression analysis revealed no enrichment or depletion using the applied isolation procedure. We presented the calculated data related to GAPDH based on these results and because GAPDH has been used as an endogenous control in several other studies of mouse brain development [15]. Moreover, GAPDH can also be used as an endogenous control in western blotting. The depletion of astrocyte marker glial fibrillary acidic protein (GFAP), pericyte marker platelet-derived growth factor beta (PDGFRb) and neuronal marker enolase 2 (Eno2) was calculated to verify endothelial cell enrichment using the isolation procedure, and these markers confirmed the usability of the isolated endothelial samples (S2 Fig).

### Western Blotting

Brain samples were suspended in RIPA buffer (80 mg/500 μL, supplemented with protease and phosphatase inhibitor cocktails (complete ULTRA tablets, Mini, EASYpack, REF05892970001; PhosSTOP, REF04906837001, Roche) and homogenized on ice using a Douncer (2 mL volume, 8 mm diameter plunger, IKA RW 14 basic rotor, IKA, Staufen, Germany) at 750 rpm, 10 times for 10 seconds. Supernatants were collected after centrifugation at 2000×*g* and 4°C for 10 minutes. The protein concentrations of the supernatants were determined using a Pierce BCA assay (Thermo Scientific, Bremen, Germany) and a bovine serum albumin (BSA) standard curve (Albumin Standard, Thermo Scientific). Before storage at −80°C, 4× Laemmli buffer supplemented with 6% β- mercaptoethanol was added to the samples. Protein (40–80 μg) and 2 μL peqGOLD prestained protein marker V (REF 27–2210, PEQLAB, Erlangen, Germany) per lane were loaded onto 7.5 or 12% sodium dodecyl sulfate–polyacrylamide gel electrophoresis gels (1.5 mm thick) after ultrasound treatment and a 5-minute denaturation at 70°C. After gel electrophoresis at 130 V, proteins were immunoblotted onto polyvinylidene difluoride membranes (162–0177, Biorad, München, Germany) in a tank blotter at 40 mA per gel at 4°C overnight. Membranes were blocked with 5% milk powder in PBS for 1 hour, and primary antibodies were applied in a 0.5% BSA/PBS solution at 4°C overnight. The primary antibodies are listed in S2 Table. Membranes were washed three times with 0.1% Tween 20/PBS for 10 minutes and blocked further with 5% milk powder/PBS for 25 minutes at room temperature (RT). Membranes were incubated with a secondary horseradish peroxidase (HRP)—labeled antibody solution (secondary antibodies are listed in the S2 Table) at RT for 1 hour and washed three times with 0.1% Tween 20/PBS. Western blots were incubated with ECL solutions for 3 minutes and developed using a FluorChem FC2 Multiimager II (Alpha Innotech, Hessisch Oldendorf, Germany) to visualize the protein bands. The density values of single protein bands were calculated using the Alpha View software using background substraction and related to the corresponding GAPDH bands.

### Immunostaining

Optimal cutting temperature (OCT) solution-embedded tissues were sectioned at 20 μm on a cryostat (Microm HM550 OMVP, Thermo Scientific), collected on microscope slides (J1800AMNZ, Thermo Scientific) and stored at -20°C until used. Slices were defrosted at 37°C and rehydrated in PBS. Tissue was fixed in -20°C methanol at RT for 10 minutes and subjected to permeabilization/blocking buffer (20% NGS, 0.01% Triton X-100 in sterile PBS) for one hour to block unspecific binding sites. Primary antibodies against claudin-5 and platelet endothelial adhesion molecule (Pecam)-1/CD31 were diluted in 0.5% BSA/0.25% Triton X-100/PBS (pH = 7.2), and the slides were incubated over night at 4°C. Slides were washed six times for 5 minutes in PBS and incubated with the respective secondary antibody solution (0.5% BSA/0.25% Triton X-100/PBS (pH = 7.2) for one hour at RT (for the antibodies, see S2 Table). The previous and following steps were performed in the dark. After six washing steps in PBS, DAPI was applied for 10 minutes, and the slides were post-fixed for five minutes in 4% PFA on ice, washed in PBS and mounted with AquaPolymount. Analysis was performed using a Nikon Eclipse 80i Fluorescence-Microscope connected to a Nikon Digital Sight DS-Qi1 camera. Confocal microscopy was performed when indicated using a Nikon C1*si* scanhead attached to an Eclipse T2000 E inverted microscope. Specifically, z-stacks of 20μm thick sections were scanned with a step size of 4μm and a 2D maximum intensity projection (MIP) image was generated. MIP pictures were analyzed for vessel length and number via Pecam-1 staining using the NIS-Elements software.

### Statistics

Statistical analysis was performed using the Sigma Plot 11.0 Statistical Software package (Systat Software, Erkrath, Germany). For each experimental group, brains from at least 3 different litter were analyzed and compared to the according NaCl control group. qPCR method development revealed that only samples should be compared to each other which have undergone experimental treatment, brain isolation, storage, purification and analysis preparation steps together. Therefore, for each DEX-treatment the according NaCl control group was done at the same time. Moreover, due to the large total number of samples, but limited sample number which could be purified at the same time, only samples from mice at the same age and same number of antenatal injections were compared to each other by using a two-tailed Student`s *t-test*. Data are presented as the means ± SEM.

## Results

### Influence of antenatal dexamethasone treatment on tight junction expression in pup brains

**Fig 1:**
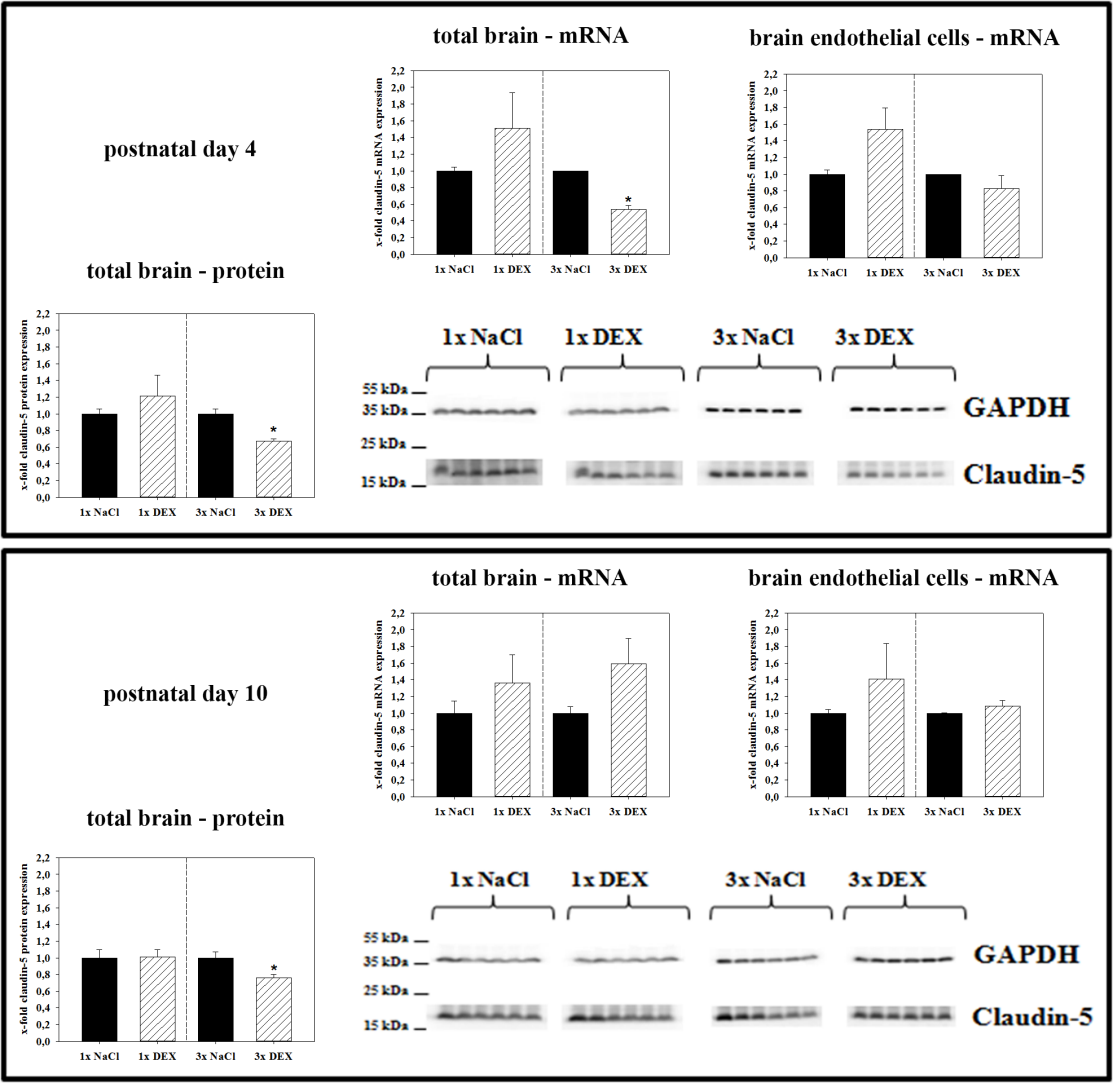
Effects of antenatal DEX treatment on claudin-5 expression in total brains and brain endothelial fractions at PN4 and PN10 pups. Data are presented as the means ± SEM. For mRNA data: n = 5–6 biological samples. At PN4, 2–3 brains from one litter were pooled to one biological sample; at PN10, one brain represented one biological sample. For western blotting: n = 6; at PN4 and PN10, one brain represented one biological sample. Biological samples were collected from at least three different litters; claudin-5 data were normalized to the expression of the endogenous control GAPDH, samples from antenatally dexamethasone treated mice were compared to corresponding samples from NaCl treated mice, *: p<0.05 (two-tailed Student’s *t-test*).

The major tight junction molecule and brain endothelial cell marker claudin-5 was investigated initially. Triple maternal DEX treatment significantly reduced claudin-5 mRNA expression to 0.54 ± 0.04-fold (p<0.05) in total brains and 0.83 ± 0.15-fold (not significant, p = 0.35) in isolated brain endothelial cells of PN4 pups compared with controls (Fig 1). Significant depletion of markers for astrocytes (GFAP), pericytes (PDGFb) and neurons (Eno2) confirmed the usability of isolated brain endothelial fractions (S2 Fig). However, it has to be mentioned that brain endothelial fractions could also contain endothelial cells from brain venules and arterioles. In addition, a minimal contamination with CNS cells (especially pericytes and astrocytes) of the isolated brain endothelial cell fraction can not be excluded using the applied dynabeads based preparation technique. With regared to claudin-5 mRNA expression, no significant regulation was detected in PN4 brain samples after single antenatal DEX treatment nor in any of the PN10 samples. Western blotting from total brain samples confirmed the regulation of claudin-5 at the protein level, which was consistent with the qPCR results. Triple DEX treatment significantly decreased claudin-5 expression to 0.67 ± 0.03-fold (p<0.05) compared with the controls, whereas single DEX treatment increased claudin-5 protein expression in the brains of PN4 pups in a non-significant manner. Interestingly, at PN10 triple antenatal DEX treatment led to a weaker, but persistent, claudin-5 reduction to 0.76 ± 0.04-fold compared with PN4.

**Fig 2:**
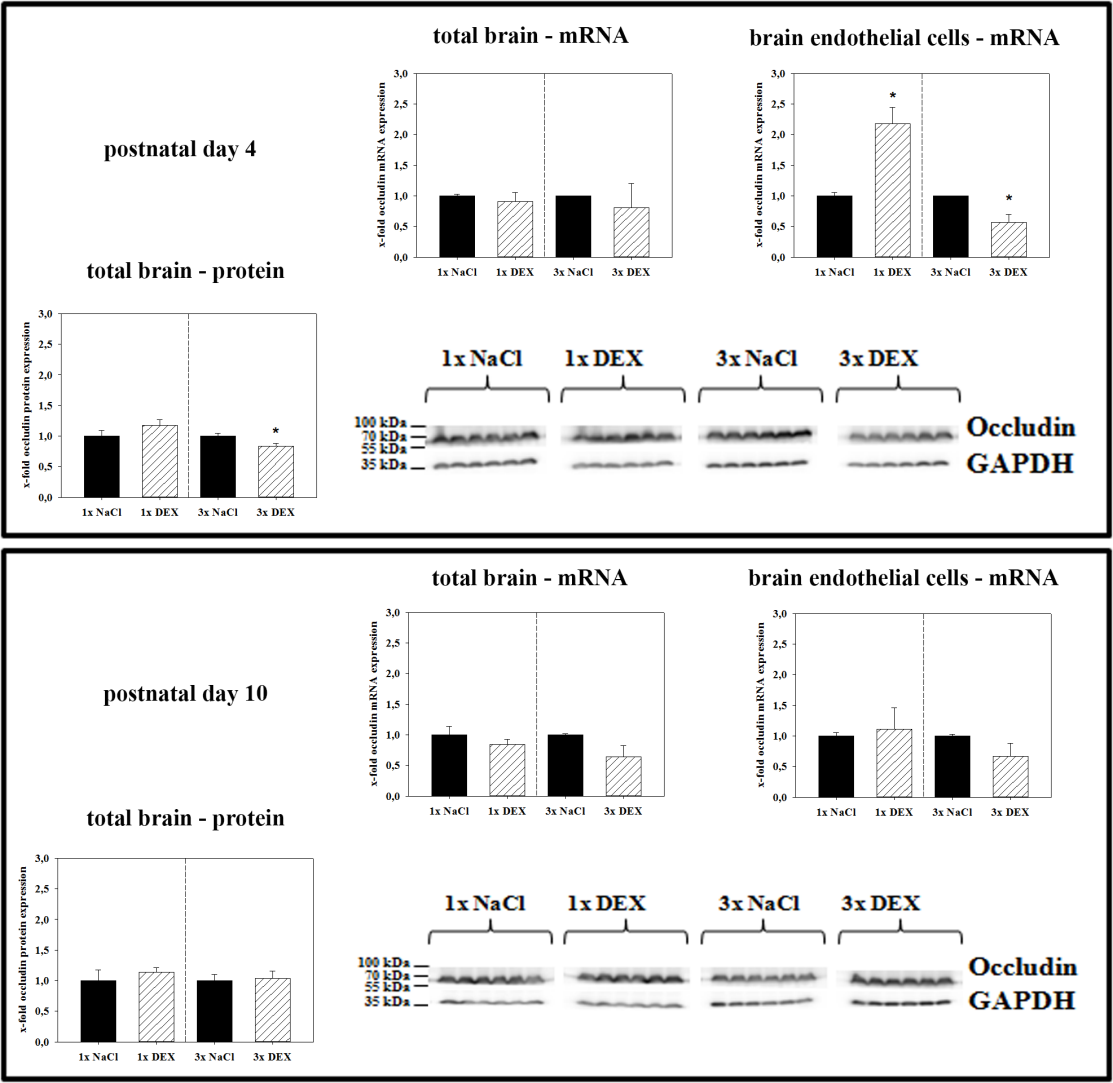
Effects of antenatal DEX-treatment on occludin expression in total brains and brain endothelial fractions of PN4 and PN10 pups. Data are presented as the means ± SEM. For mRNA data: n = 5–6 biological samples; at PN4, 2–3 brains from one litter were pooled to one biological sample; at PN10, one brain represented one biological sample. For western blotting, n = 6; at PN4 and PN10, one brain represented one biological sample. Biological samples were collected from at least three different litters; data were normalized to the expression of the endogenous control GAPDH, samples from antenatally dexamethasone treated mice were compared to corresponding samples from NaCl treated mice, *: p<0.05 (two-tailed Student’s *t-test*).

The expression of the second major tight junction molecule, occludin, which is a confirmed dexamethasone target gene [16], was also investigated. In contrast to claudin-5, single DEX treatment upregulated occludin expression at the mRNA level in PN4 isolated brain endothelial cells to 2.18 ± 0.26-fold (p<0.05, Fig 2). Moreover, triple DEX treatment reduced occludin mRNA expression in isolated brain endothelial cells to 0.57 ± 0.13-fold (p<0.05) compared with the controls, whereas no changes of occludin mRNA expression were detected in total brain PN4 samples after single or triple DEX treatments. At PN10 no significant changes in occludin mRNA expression were found. In concordance with mRNA data, triple DEX-treatment significantly decreased the occludin protein levels to 0.83 ± 0.05-fold in total PN4 brains (p<0.05). However, no changes in PN4 brains were found after a single DEX treatment or generally in PN10 brains, regardless of the treatment regimen.

**Fig 3:**
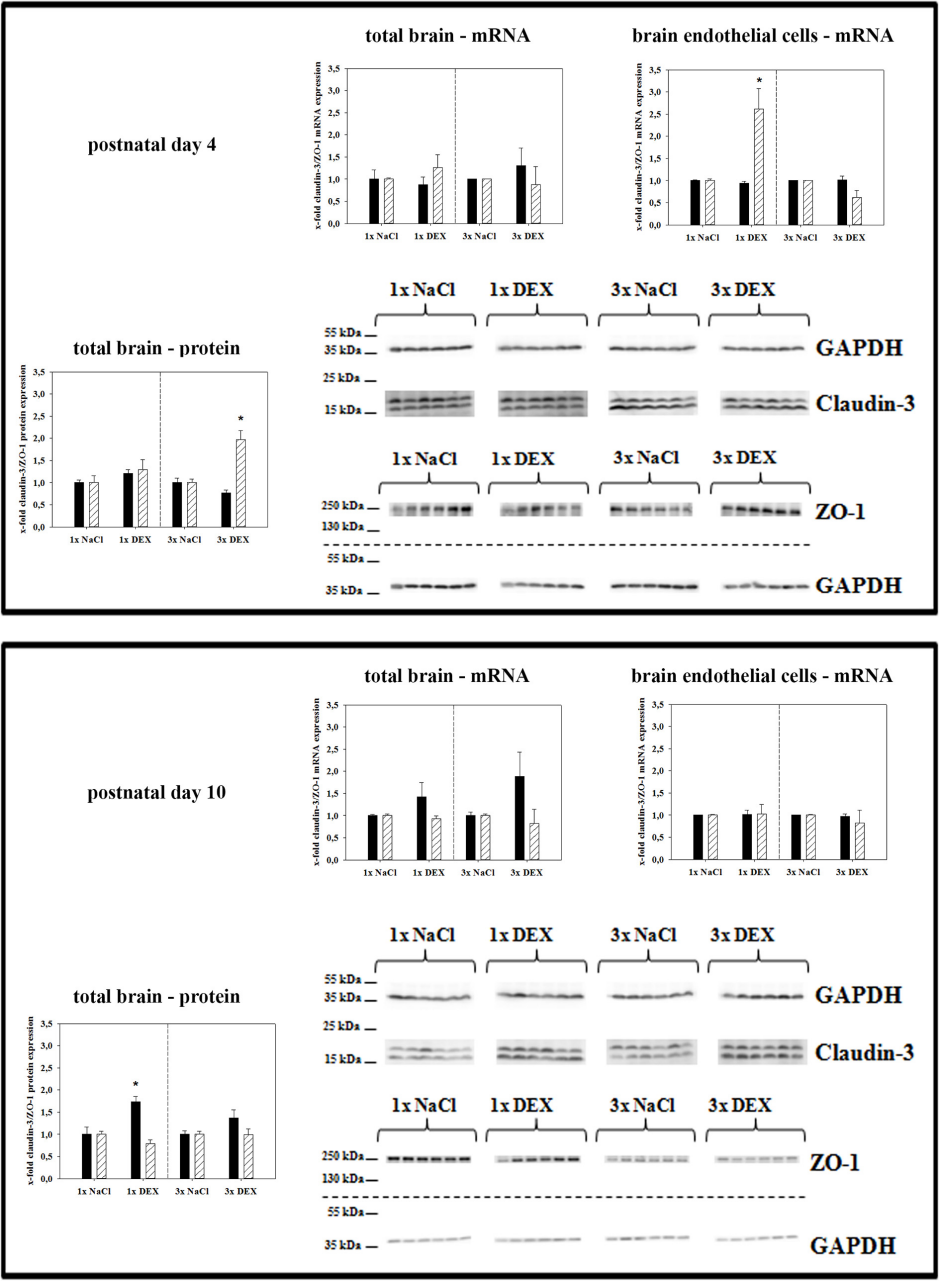
Effects of antenatal DEX-treatment on claudin-3 (black bars) and ZO-1 (white bars) expression in total brains and brain endothelial fractions of PN4 and PN10 pups. Data are presented as the means ± SEM. For mRNA data, n = 5–6 biological samples; at PN4, 2–3 brains from one litter were pooled to one biological sample; at PN10, one brain represented one biological sample. For western blotting, n = 6; at PN4 and PN10, one brain represented one biological sample. Biological samples were collected from at least three different litters; data were normalized to the expression of the endogenous control GAPDH, samples from antenatally dexamethasone treated mice were compared to corresponding samples from NaCl treated mice, *: p<0.05 (two-tailed Student’s *t-test*).

The expression of claudin-3 and zonula occludens-1 (ZO-1) was also analyzed, and the results are summarized in Fig 3. Notably, no significant changes were found for claudin-3 expression at PN4, whereas its protein expression was upregulated to 1.73 ± 0.12 (p<0.05) in total PN10 brains after single DEX treatment. In case of ZO-1, single DEX treatment increased mRNA expression in isolated brain endothelial cells to 2.62 ± 0.47-fold (p<0.05). In general, the regulation pattern of ZO-1 was similar to that of occludin.

### Influence of antenatal dexamethasone treatment on receptor expression in pup brains

**Fig 4:**
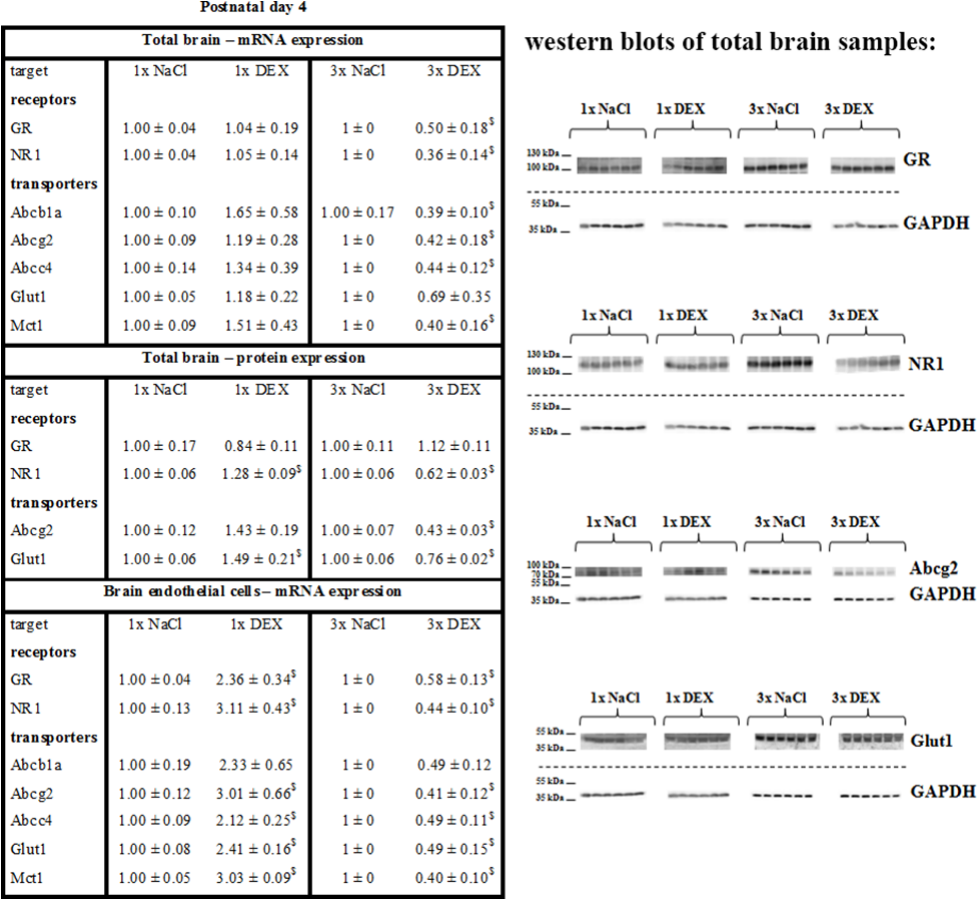
Effects of antenatal DEX-treatment on receptor and transporter expression in total brains and brain endothelial fractions of PN4 pups. Data are presented as the means ± SEM. For mRNA data, n = 5–6 biological samples; at PN4 2–3, brains from one litter were pooled to one biological sample. For western blotting, n = 6; at PN4, one brain represented one biological sample. Biological samples were collected from at least three different litters; claudin-5 data were normalized to the expression of the endogenous control GAPDH, samples from antenatally dexamethasone treated mice were compared to corresponding samples from NaCl treated mice, $: p<0.05 (two-tailed Student’s *t-test*).

The expression of the glucocorticoid receptor (GR) and NR1 subunit of N-Methyl-D-aspartate (NMDA) receptor was investigated after maternal DEX treatments. The results are summarized in Fig 4. GR and NR1 mRNA expression did not change after single DEX treatment in total PN4 brains, but both receptors were downregulated significantly after triple maternal DEX treatment (GR: 0.50 ± 0.18-fold; NR1: 0.36 ± 0.14-fold, p<0.05). GR and NR1 mRNA were upregulated in brain endothelial fractions after single maternal DEX treatment (GR: 2.36 ± 0.34-fold, NR1: 3.11 ± 0.43-fold, p<0.05), but downregulated after triple maternal DEX treatment (GR: 0.58 ± 0.13-fold, NR1: 0.44 ± 0.10-fold, p<0.05) at PN4. The NR1 protein levels significantly increased after single maternal DEX treatment to 1.28 ± 0.09-fold (p<0.05), but its expression decreased in the triple maternal DEX treatment group to 0.62 ± 0.03-fold in total PN4 brains (p<0.05). No significant regulation of the GR protein level was found. At PN10 no significant changes in the mRNA expression of GR or NR1 were determined regardless of DEX treatment (see S3 Fig). NR1 protein expression remained decreased (0.69 ± 0.04-fold, p<0.05) after triple maternal DEX treatment in total PN10 brains.

### Influence of antenatal dexamethasone treatment on transporter expression in pup brains

The primary ATP-binding cassette (ABC) transporters of the BBB that are relevant for multidrug resistance, such as Abcb1a (P-glycoprotein), Abcc4 (Mrp4) and Abcg2 (Bcrp), were investigated. Monocarboxylate transporter 1 (Mct1, Slc16a1) and glucose transporter 1 (Glut1, Slc2a1) were also examined due to their important role in metabolism and energy balance. Moreover, Glut1 is an established marker of brain endothelial cells. The results are summarized in Fig 4. The mRNA expression levels of Abcb1, Abcg2, Abcc4 and Mct1 decreased after triple maternal DEX treatment in PN4 total brains (Abcb1a: 0.39 ± 0.10-fold; Abcg2: 0.42 ± 0.18-fold; Abcc4: 0.44 ± 0.12-fold; Mct1: 0.40 ± 0.16-fold, p<0.05). Single antenatal DEX treatment significantly increased the expression of Abcg2 (3.01 ± 0.66-fold, p<0.05), Abcc4 (2.12 ± 0.25-fold, p<0.05), Mct1 (3.03 ± 0.09-fold, p<0.05) and Glut1 (2.41 ± 0.16-fold, p<0.05) in brain endothelial fractions, but triple antenatal DEX treatment significantly decreased the mRNA expression of all transporters to values similar to those of total brains at PN4. Abcg2 and Glut1 protein expression was analyzed due to their high abundance. Western blotting of PN4 brain samples revealed an increased amount of Glut1 (1.49 ± 0.21-fold, p<0.05) protein after single maternal DEX treatment and a decreased expression of Abcg2 (0.43 ± 0.03-fold, p<0.05) and Glut1 (0.76 ± 0.02-fold, p<0.05) protein after triple maternal DEX treatment. The expression changes adapted to the control group values at PN10 with some exceptions (mRNA total brain: Abcg2 (0.92 ± 0.02-fold, p<0.05, 1x DEX), Abcb1a (0.60 ± 0.11-fold, p<0.05, 3x DEX); brain endothelial cell fractions: Abcc4 (0.49 ± 0.14-fold, p<0.05, 3x DEX); protein total brain: Glut1 (0.75 ± 0.03-fold, p<0.05, 3x DEX); see S3 Fig).

### Immunofluorescence microscopic analysis

**Fig 5:**
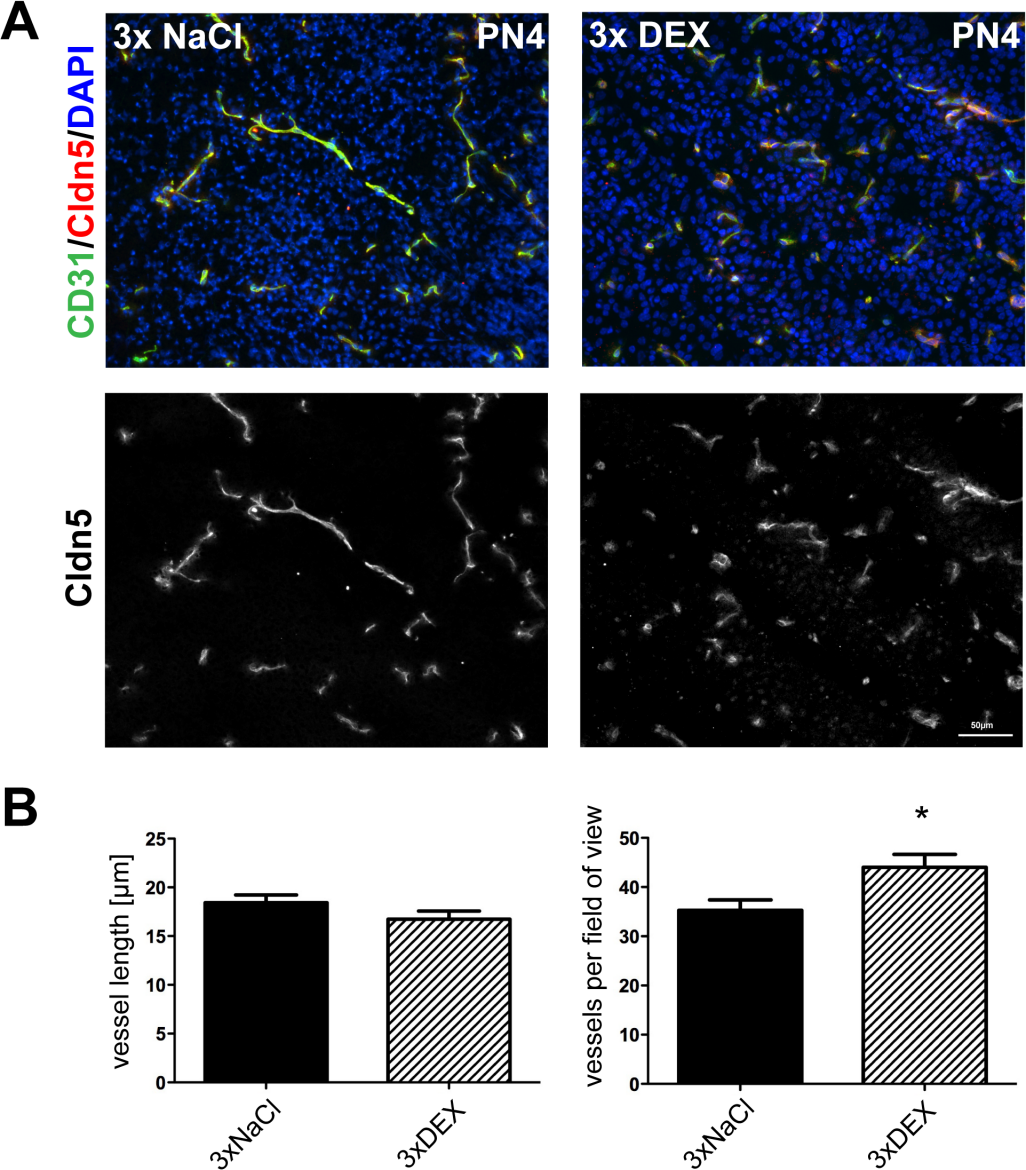
Effects of triple antenatal DEX-treatment on vessel morphology of PN4 pups. Representative immunofluorescence images of brain slices obtained from PN4 pups treated antenatally either with 3x NaCl or 3x DEX. Merged images of claudin-5 (Cldn5) (red), Pecam-1/CD31 (green) and DAPI (blue) were presented. The bar indicates a length of 50 μm. Images were magnified 20x (A); the average vessel length was measured, and the number of vessel per field of view was counted, revealing that antenatal 3x DEX treatment led to more, but shorter, brain vessels in comparison with brains of 3x NaCl-treated control animals (B), n = 6 per treatment, *: p<0.05 (two-tailed Student’s *t-test*).

PN4 samples were analyzed using immunofluorescence microscopy because the most prominent effects were found in PN4 brains after triple DEX treatments. Immunofluorescence images of claudin-5 and Pecam-1/CD31 were generated and analyzed to localize and quantify changes in endothelial marker expression (Fig 5). Image analysis revealed changes in vessel morphology. Triple antenatal DEX treatment significantly increased the number of vessels per section compared with the triple NaCl-control (44.03 ± 2.61 vs. 35.25 ± 2.14, mean ± SEM, n = 6, p<0.05), but the average vessel length was in tendency reduced after triple DEX treatment (16.74 ± 0.82 μm vs. 18.42 ± 0.79 μm, mean ± SEM, n = 6, non-significant, p = 0.17). However, no significant difference in total areas of vascularization were observed.

### Possible mechanisms

Comparisons of the expression data of total brain samples with the brain endothelial fractions of PN4 pups from triple dexamethasone-treated mothers as well as corresponding immunofluorescence images suggested altered vascularization. Therefore, the mRNA expression of angiogenic-relevant molecules, such as vascular endothelial growth factor-A (VEGF-A), vascular endothelial growth factor receptor 2 (VEGFR2), neuropilin-1 (Nrp1), Tie-2, angiopoietin-1 (angpt1) and angiopoietin-2 (angpt2), were investigated using qPCR. The results revealed a significant downregulation of all investigated molecules in total brain samples and endothelial fractions (Table 1). For example, VEGF-A and VEGFR2 expression decreased to 0.48 ± 0.17-fold (p<0.05) and 0.47 ± 0.13-fold (p<0.05), respectively, after triple antenatal DEX treatment at PN4 in total brains. This was consistent with their reduced expressions of 0.61 ± 0.13-fold (p<0.05) and 0.46 ± 0.13-fold (p<0.05), respectively, in the brain endothelial cell fractions. No significant changes were found in total PN4 brains in single maternal antenatal DEX treatment, but an upregulation of VEGF-A in the brain endothelial fraction to 2.20 ± 0.25-fold (p<0.05) was detected. The expression changes of other angiogenic markers, such as neuropilin-1, Tie-2, angiopoietin-1 and angiopoietin-2, exhibited a similar picture at PN4. In particular, the reduced marker expression in brain endothelial cell fractions led to the hypothesis of reduced angiogenic activity in the brains of PN4 pups whose mothers had been treated three times with DEX.

**Table 1:** Changes of mRNA expression of mechanistic brain markers of PN4 mouse pups by triple antenatal DEX-treatments.

Postnatal day 4				
**Total brain–mRNA expression**				
Target	1x NaCl	1x DEXl	3x NaCl	3x DEX
**Angiogenesis**				
VEGF-A	1.00 ± 0.13	0.97 ± 0.19	1 ± 0	0.48 ± 0.17^$^
VEGFR2	1.00 ± 0.14	1.14 ± 0.33	1 ± 0	0.47 ± 0.13^$^
Nrp1	1.00 ± 0.09	1.16 ± 0.23	1 ± 0	0.37 ± 0.12^$^
Tie-2	1.00 ± 0.15	1.20 ± 0.40	1 ± 0	0.33 ± 0.09^$^
Angpt1	1.00 ± 0.01	1.02 ± 0.17	1 ± 0	0.43 ± 0.17^$^
Angpt2	1.00 ± 0.11	1.07 ± 0.22	1 ± 0	0.37 ± 0.16^$^
**Shh-pathway**				
Shh	1.00 ± 0.007	1.12 ± 0.20	1 ± 0	0.37 ± 0.17^$^
PTCH1	1.00 ± 0.05	0.91 ± 0.12	1 ± 0	0.56 ± 0.25
Sox-18	1.00 ± 0.05	1.28 ± 0.20	1 ± 0	0.54 ± 0.13^$^
**Wnt-pathway**				
Axin-2	1.00 ± 0.003	0.94 ± 0.19	1 ± 0	0.45 ± 0.15^$^
**Brain endothelial cells–mRNA expression**				
Target	1x NaCl	1x DEXl	3x NaCl	3x DEX
**Angiogenesis**				
VEGF-A	1.00 ± 0.09	2.20 ± 0.25^$^	1 ± 0	0.61 ± 0.13^$^
VEGFR2	1.00 ± 0.17	1.92 ± 0.57	1 ± 0	0.46 ± 0.13^$^
Nrp1	1.00 ± 0.03	3.01 ± 0.27^$^	1 ± 0	0.51 ± 0.12^$^
Tie-2	1.00 ± 0.24	2.96 ± 0.39^$^	1 ± 0	0.43 ± 0.13^$^
Angpt1	1.00 ± 0.02	3.53 ± 0.64^$^	1 ± 0	0.57 ± 0.13^$^
Angpt2	1.00 ± 0.09	2.50 ± 0.50^$^	1 ± 0	0.31 ± 0.07^$^
**Shh-pathway**				
Shh	1.00 ± 0.11	3.41 ± 0.32^$^	1 ± 0	0.66 ± 0.19
PTCH1	1.00 ± 0.02	2.32 ± 0.13^$^	1 ± 0	0.57 ± 0.13^$^
Sox-18	1.00 ± 0.06	2.09 ± 0.24^$^	1 ± 0	0.85 ± 0.36
**Wnt-pathway**				
Axin-2	1.00 ± 0.05	1.99 ± 0.21^$^	1 ± 0	0.54 ± 0.12^$^

mRNA expression of mechanistic brain markers of PN4 mouse pups changed by triple antenatal DEX-treatments. Data are presented as the means ± SEM; n = 5–6 biological samples, at PN4 2–3 brains from one litter were pooled to one biological sample, biological samples were collected from at least three different litters

$: p<0.05 after two-tailed Student’s *t-test*.

Recently, it was shown that the sonic hedgehog pathway (Shh) plays an important role in the barrier function of the brain endothelium [17]. Consequently, we were interested in investigating this pathway. The results showed a significant decrease in Shh and SRY (sex determining region Y) box 18 (Sox-18) in total PN4 brain samples and PTCH1 in brain endothelial cells after triple DEX treatment. However, the expression of Shh and Sox-18 was not significantly changed in the brain endothelium (Table 1). Sox-18 directly controls the transcription of claudin-5. Therefore, the non-affected Sox-18 expression is consistent with the unchanged claudin-5 expression in brain endothelial cells of PN4 pups after a three-time dexamethasone treatment (comparison with the data in Fig 1).

We additionally analyzed the expression of Wnt-pathway target Axin-2 because claudin-3 expression was differently regulated compared with claudin-5 and because claudin-3 was associated with brain development and regulation by the Wnt-pathway [18]. A significant decrease in Axin-2 to 0.45 ± 0.15-fold (p<0.05) compared with vehicle-treated animals was observed after triple antenatal DEX treatment in total PN4 brains, which confirmed a possible modulating action of the Wnt-pathway. Axin-2 was also downregulated in brain endothelial cell fractions after triple antenatal DEX treatment at PN4, which is similar to other markers. In summary, triple antenatal DEX treatment affected the expression of markers of angiogenesis, sonic hedgehog and Wnt-pathway, which suggests a complex interplay among these mechanisms in brain development.

## Discussion

Pregnant women who are at risk of preterm delivery are commonly treated with synthetic glucocorticoids (GCs) to ensure the lung maturation and survival of the preterm infant [2,3]. Usually, GCs are administered to pregnant mothers in a single course. However, they may receive repeated GC courses until the birth of the infant, despite little evidence that repeated courses are more beneficial than single courses. Multiple courses could lead to impairments of respiratory adaptions early before birth and, in relation to brain development, could reduce motor skills, learning and memory performance and lead to cerebral palsy [19]. One feature of cerebral palsy is white matter lesions, also called periventricular leukomalacia, which are associated with myelin loss. Decreased myelination and brain cell proliferation has been described after multiple antenatal GC courses and was accompanied by a decelerated maturation of oligodendrocyte progenitor cells [20]. However, data on the role of the cerebral vasculature during this process are rarely available. It has been assumed that functional vascular wall immaturity can also be a factor in vulnerability to white-matter damage, but no comprehensive investigation of the involved molecular targets and mechanisms at the BBB has been published [21]. Consequently, our work established a mouse model to study the effects of single and repeated dexamethasone (DEX) application on blood-brain barrier (BBB) properties. Mice were chosen as a model because their lung and brain development is delayed compared with those of humans [22]. This advantage enables mouse mothers to deliver their pups regularly in the experimental setting in contrast to, e.g., sheep. Brain developmental stages of sheep are close to humans and analysis of pre-term pups would require surgical interventions. In the presented study brains of mouse pups were investigated from postnatal day four (PN4) and 10 (PN10) that can be used as models for the developmental status of pre-term and term human newborns, respectively [14].

Tight junction proteins are essential for the establishment of the physical barrier of the blood-brain barrier by sealing the intercellular gaps between brain endothelial cells. Loss of tight junctions is associated with a disrupted BBB causing an imbalance of homeostasis within the brain [8,9]. Therefore, we analyzed the expression of tight junction molecules in the brain. Claudin-5 is currently believed to be the major claudin at the BBB that is responsible for barrier functionality [9]. In our model, triple DEX treatment significantly reduced claudin-5 expression in total brain samples of PN4 pups at the mRNA and protein levels.

In concordance to this, Sadowska *et al*. (2009) found upregulated claudin-5 in the cerebral cortex in sheep fetuses after a single DEX-course, but no effects on claudin-5 expression in the cortex after multiple DEX-courses. Basically, this accords with a relative decrease after the multiple DEX-treatment in comparison to the single course [11]. Interestingly, we found no significant change in mRNA expression of claudin-5 in isolated mouse brain endothelial cells after triple DEX-treatment indicating a total loss of claudin-5 protein in the brain, but not per endothelial cell. Analysis of PN10 pups showed less significant effects on claudin-5 expression. This suggested that brain development stages of the pups treated with dexamethasone assimilated within time which was recently shown also for rats [23].

In case of occludin, an analysis of isolated brain endothelial cells revealed a distinct upregulation after a single DEX course and a more significant downregulation to 0.57-fold after a triple DEX application, whereas almost no significant changes were found in PN4 total brain samples. In this context, it has to be kept in mind that occludin in the brain may be expressed not only by brain capillary endothelial cells but also by pericytes and subtypes of neurons, in contrast to claudin-5 [24,25]. Occludin is a direct target gene of the GC receptor in mouse and can be activated by dexamethasone [16]. These facts may explain the detection of only small differences in total brain samples, whereas isolated brain endothelial cell fractions highlighted and confirmed occludin regulation at the BBB. In comparison with our data, Sadowska *et al*. (2009) showed an upregulation of occludin in the cerebellum, but not in the cerebral cortex, of sheep fetuses after a single DEX course [11]. After multiple DEX-courses they also found an upregulation of occludin, but this time in the cerebral cortex and the cerebellum [11]. Differences between results of our mouse model and the sheep model after multiple DEX courses could be explained by species differences and different experimental settings, especially the possibility of recovery phases after weekly DEX-treatments in the sheep model (in contrast to daily injections in the mouse model) considering the short biological half-life of DEX of about 36 hours. Furthermore, we analyzed samples from the total brain, whereas the work in the sheep models clearly demonstrated that expression changes of tight junction molecules after antenatal maternal DEX courses are brain region specific [11,12]. However, qPCR of isolated brain endothelial fractions derived from total brain samples confirmed DEX-effects on occludin expression at the BBB level. This was concordant to several *in-vitro* studies with brain endothelial cells, where GCs (dexamethasone, hydrocortisone) were able to upregulate expression of tight junction proteins and increase barrier tightness [15,26,27]. In case of tight junction associated protein ZO-1, the expression was increased in the cerebral cortex after repeated DEX courses in the sheep model [11]. Interestingly, we also found ZO-1 protein upregulation after repeated DEX injections in the PN4 samples (Fig 3). The differential regulation of tight junction proteins claudin-5, occludin and ZO-1 in our model is an example for the very complex tight junction network, in which every single protein has its own function. Thus, our findings of claudin-5 and occludin downregulation and ZO-1 upregulation after antenatal, triple DEX treatment on PN4 are not contradictory. Moreover, ZO-1 total protein expression data do not necessarily reflect ZO-1 functionality in the cellular membrane. In this context, it was recently shown that ZO-1 protein expression in brain endothelial cells was upregulated in an *in-vitro* stroke model although the BBB was damaged [28]. In this case, further experiments elucidated that ZO-1 localization became more discontinuous in the cellular membrane which was in concordance with the breakdown of the barrier. Therefore, future studies are necessary to clarify to which extent ZO-1 total protein upregulation by antenatal triple DEX treatment correlates to its function within the tight junction network and the cellular membranes.

Because DEX upregulates occludin via the glucocorticoid receptor (GR) at the BBB, we analyzed changes in GR expression after different antenatal DEX regimens. Several studies have shown that GR expression decreased after activation by GCs [27]. In our case, sustained effects on GR expression in total brain samples were only detected after triple DEX treatment at the mRNA level (0.5-fold). Analyses of brain endothelial cell fraction revealed that single DEX treatment increased GR mRNA significantly (2.36-fold), whereas triple DEX treatment decreased its expression (0.58-fold). These data confirmed that regulations at the BBB level may be overlooked when only total brain samples are investigated. To interpret the data from the total brain samples, it must be considered that GR is also found in neurons or astrocytes [29,30]. The expression of the subunit NR1 of the NMDA receptor was also studied. NMDAR occurs in neurons and astrocytes as well as brain endothelial cells [10]. NMDAR plays a pivotal role in learning, long-term potentiation and synaptic plasticity in the CNS, and subunit composition changes during development [31]. The role of NMDAR in brain endothelial cells remains to be elucidated. Some publications have suggested the importance of this receptor in signaling pathways linked with cyclooxygenases and prostaglandins, particularly in diseases such as epilepsy, multiple sclerosis or stroke [32]. Owen *et al*. (2004) showed a decrease in NR1 expression after repeated DEX treatment in female guinea pig brains [33]. In concordance with these results, in our model NR1 was significantly downregulated in total brain samples after antenatal triple DEX treatments at the mRNA and protein levels. In the isolated brain endothelial cell fraction, NR1 was highly upregulated after a single DEX course and downregulated after a triple DEX course (Fig 4). A huge array of transporters is expressed at the BBB to regulate the influx and efflux of solutes to the CNS. In particular, ABC transporters such as Abcb1a, Abcg2 and Abcc4 are considered a first defense line at the BBB against unwanted substances. These transporters are also relevant for the phenomenon of multidrug-resistance [34]. The altered expression of these transporters most likely also changes the susceptibility of drugs to the CNS. Maternal antenatal triple DEX treatments resulted in a significant reduction of mRNA expression of Abcb1a, Abcg2 and Abcc4 in total brain PN4 samples, which was confirmed at the BBB level. Notably, an upregulation at the BBB level was observed after a single DEX course. The expression of glucose transporter Glut1 and lactate transporter Mct1 was changed in a similar manner.

In general, differences in BBB marker expression (claudin-5) at PN4 in brain endothelial versus total brain samples after triple DEX treatment suggested changes in the morphology of brain vessels. In concordance with the BBB markers, the expression of astrocyte (GFAP), pericyte (PDGFRb) and neuronal (Eno2) markers was significantly reduced by antenatal triple DEX treatments at PN4, which confirmed that other cellular partners of the neurovascular unit were affected (S3 Table). In this regard, immunofluorescence images of PN4 samples revealed that triple antenatal DEX treatment led to a significantly changed vessel morphology. In concordance with our data, it was recently shown that the excessive DEX treatment of pregnant mice daily from E11 to E17 led to a significant decrease in the average capillary length of the paraventricular nucleus of the hypothalamus by 13% at PN20 [13]. Furthermore, the authors have also shown brain region specific effects of excessive DEX treatment on FITC leakage by which they described BBB tightness. This was in line with brain region specific effects of DEX treatment on tight junction protein expression reported in sheep models.^11^ A decreased expression of tight junction molecules such as claudin-5 was directly correlated to paracellular leakiness [35,36]. Thus, it could be assumed that multiple DEX courses in our model led to increased paracellular leakage on day PN4 since protein expression of claudin-5 and occludin were decreased in total brain. However, isolated brain endothelial fractions did not reveal a significant downregulation of claudin-5 mRNA indicating the possibility of changed capillary morphology with a still intact BBB. In this context, premature infants also showed evidence of physiological immaturity in the cerebral vasculature in immature white matter [37]. Therefore, in order to resolve this, further systematic studies are needed to investigate the correlation of tight junction protein expression in brain endothelial cells (and not total brain) and BBB tightness including brain region specifity at several different time points. A limitation for a brain region specific protein analysis of the brain endothelial fraction for early time-points such as in our case at PN4 could be the efforts to collect enough tissue.

Then, we investigated the possible underlying molecular mechanisms for these morphological vessel alterations. First, angiogenic markers were tested, and all of the markers (VEGF-A, VEGFR2, Nrp1, Tie-2, Angpt1, Angpt2) were downregulated in total brain PN4 samples after repeated DEX courses. In this regard, it was previously shown that DEX reduced VEGF-A expression in rat placenta and impaired placental vasculogenesis [38]. In contrast to the triple DEX treatment, single DEX treatment upregulated VEGF-A in our model, which agreed with the results of another study, in which antenatal DEX treatment of in total 0.06 mg DEX/kg body weight in C57Bl/6 mice increased placental VEGF-A in females fetuses, thereby supporting a concentration-dependent DEX effect on VEGF-A regulation [39]. The downregulation of angiogenic genes such as VEGF-A would suggest reduced angiogenesis. On the contrary, results of immunofluorescence images (Fig 5) showed a slight, but significant increase in vessels per view, a tendency of a decreased vessel length, but no change in the total area of vascularization. Therefore, we concluded that antenatal, triple DEX treatment led to an altered brain vessel differentiation in PN4 mouse pups, but based on our data we could not state that a reduced angiogenesis was proven. In this regard, several facts have to be considered. First, the over all angiogenic response of the vessels is ultimately influenced by the balance of pro- and anti-angiogenic factors. Several other pro-angiogenic molecules such as basic fibroblast growth factor (bFGF), glial-derived growth factor (GDNF), brain-derived neurotrophic factor (BDNF) and transforming growth factor beta (TGFb) or anti-angiogenic factors such as angiostatin, thrombospondin or endostatin were not analyzed in the presented work [40]. Moreover, we did not measure all factors on the protein level, which would be necessary to clearly state if triple DEX treatment promotes a pro- or anti-angiogenic microenvironment. Second, other factors such as oxygen and nutrient demands could regulate angiogenesis and microvessel density [40–42]. In this regard, we have also found a DEX induced regulation of Glut1 and Mct1 which are responsible for glucose and lactate transport across brain endothelial cells into the CNS. This could be a hint for adapted metabolic rates and demands. Third, the claudin-5 stainings appeared to be more diffuse after antenatal, triple DEX treatment (Fig 5). This was in concordance with the decreased mRNA as well as protein levels of claudin-5 and, moreover, supported an altered differentation process of the brain endothelial cells. In general, using the 2D z-stack method in our study confirmed changed brain vessel morphology, but this technique does not enable us to conclude about microvessel density. To investigate changes of microvessel density other techniques such as 3D tissue-block analysis would be recommended. Since the increased number of brain vessels in our images could theoretically also originate from an increased number of vessel incisions, we would not exclude that using 3D imaging techniques could lead to the result that antenatal, triple DEX treatment also reduced angiogenesis. However, the slight, but significant increase in vessel per field of view stands as it is and further inverstigations are required to clarify the apparent discrepancy between mRNA expression of angiogenic factors and vessel morphology.

As shown in Table 1, changes were also observed in markers of the sonic hedgehog and the Wnt-pathway. All three pathways were downregulated by triple maternal DEX courses in PN4 brains. In several recent publications, an interplay of these pathways with DEX or among each other was suggested [18, 43–45]. For example, it was proposed that sonic hedgehog acts upstream of VEGF-A by inducing its expression [44]. Relationships between GC effects and Shh were shown in lung and brain, where betamethasone counteracted the adverse effects of antenatal doses of LPS in the lung in a Shh-dependent manner, but Shh decreased GC-induced brain injury by partially upregulating an 11-Beta-Hydroxysteroid Dehydrogenase Type 2 (11betaHSD2)-dependent mechanism [43,46]. Overviewing the data, multiple DEX-treatment resulted in a downregulation of the mRNA of most targets in brain endothelial fractions at PN4 suggesting a general DEX-effect on vessel development and/or differentiation.

In summary, our study showed for the first time that repeated antenatal DEX courses changed several key properties of the BBB, such as tight junctions, transporter proteins and receptors, of PN4 mouse pups. Moreover, brain vessel morphology and signaling pathways related to angiogenesis and general development were changed. These data provide a vast array of possible molecular targets that are affected by excessive antenatal DEX exposure and may be used in future studies to elucidate the underlying mechanisms of adverse DEX effects in more detail. It is suggested that the comprehensive data set about the influence of dexamethasone on BBB relevant molecules and pathways should be evaluated and verified in *in-vitro* studies before conducting further animal studies according to the 3Rs (replacement, refinement, reduction) principles of animal testing. However, it is important to mention that effects of dexamethasone on mouse brain development were shown to be region specific and that the data in the current work were obtained from total brain samples. It has also to be considered that development in mouse is significantly compressed compared to human. Therefore, data translation may not be applicable to pregnant women given a single course or additional doses of dexamethasone during pregnancy.

## Supporting Information

S1 ARRIVE Checklist (PDF)

S1 Fig Treatment scheme. Pregnant C57Bl/6 mice were delivered on day E8 by the company and acclimatized in the animal facility until they were injected either once on day E16 or for three times on day E15, E16 and E17 with 0.1 mg dexamethasone per kg body weight.
(PDF)

S2 Fig Calculated mRNA depletion factor based on Ct-value comparison after brain endothelial cell (BEC) isolation procedure of frozen PN4 brain samples. It was shown that mRNA of GAPDH was nearly unchanged in total brain versus brain endothelial fraction samples, whereas other targets often used as endogenous controls such as 18S rRNA and b-Actin were significantly depleted. Moreover, successful depletion of astrocyte marker GFAP, neuronal marker Eno2 and pericyte marker PDGFRb by the brain endothelial cell isolation procedure was proven. Extension of the calculation of the depletion factor additionally including corresponding GAPDH Ct-values (ddCt) resulted in a 4-fold depletion of GFAP and Eno2 and a 2.5-fold depletion of PDGFRb. Data are presented as means ± SEM (n = 23), statistical significant difference to GAPDH was indicated by * (p<0.05, two-tailed Student’s *t-test*).
(PDF)

S3 Fig Effects of maternal DEX-treatment on receptor and transporter expression in total brains as well as brain endothelial fractions of PN10 pups. Data are presented as means ± SEM; for mRNA data: n = 5–6 biological samples, at PN10 one brain represented one biological sample; for western blotting: n = 6, at PN10 one brain represented one biological sample. Biological samples were collected from at least three different litters; $: p<0.05 (two-tailed Student’s *t-test*). GR = glucocorticoid receptor; NR1 = NR1 subunit of N-methyl-D-aspartate receptor; Abcb1a = ABC-transporter b1a (= P-glycoprotein); Abcg2 = ABC-transporter g2 (= bcrp); Abcc4 = ABC-transporter c4 (= Mrp4); Glut1 = glucose transporter 1 (= Slc2a1), Mct1 = monocarboxylic acid transporter 1 (= Slc16a1).
(PDF)

S1 Table List of Taqman-probes used for qPCR. (PDF)

S2 Table List of antibodies used for western blotting and immunofluorescence microscopy. (PDF)

S3 Table Effects of antenatal DEX-treatment on cell marker expression of Pecam-1 (brain endothelial cells), PDGFRb (pericytes), GFAP (astrocytes), Eno2 (neurons) in total brains as well as brain endothelial fractions of PN4 and PN10 pups. (PDF)
