# Supplementary material for: Multiple Antenatal Dexamethasone Treatment Alters Brain Vessel Differentiation in Newborn Mouse Pups
Source: PLoS One. 2015 Aug 14;10(8):e0136221. doi: 10.1371/journal.pone.0136221 (PMC4537167; doi:10.1371/journal.pone.0136221)

S1 Fig: Treatment scheme. Pregnant C57Bl/6 mice were delivered on day E8 by the company and acclimatized in the animal facility until they were injected either once on day E16 or for three times on day E15, E16 and E17 with 0.1 mg dexamethasone per kg body weight.

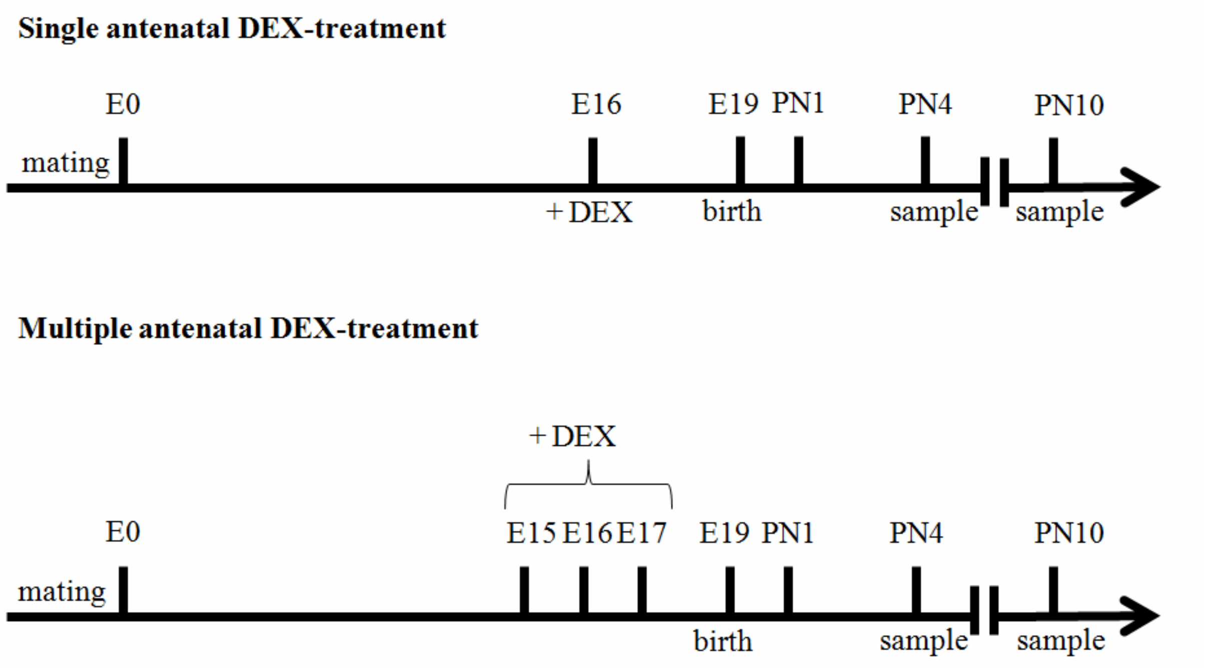

Supplement: S1 Fig — Pregnant C57Bl/6 mice were delivered on day E8 by the company and acclimatized in the animal facility until they were injected either once on day E16 or for three times on day E15, E16 and E17 with 0.1 mg dexamethasone per kg body weight. (PDF) [file pone.0136221.s002.pdf]
