# Supplementary material for: Multiple Antenatal Dexamethasone Treatment Alters Brain Vessel Differentiation in Newborn Mouse Pups
Source: PLoS One. 2015 Aug 14;10(8):e0136221. doi: 10.1371/journal.pone.0136221 (PMC4537167; doi:10.1371/journal.pone.0136221)

**S2 Fig.** Calculated mRNA depletion factor based on Ct-value comparison after brain endothelial cell (BEC) isolation procedure of frozen PN4 brain samples. It was shown that mRNA of GAPDH was nearly unchanged in total brain versus brain endothelial fraction samples, whereas other targets often used as endogenous controls such as 18S rRNA and b-Actin were significantly depleted. Moreover, successful depletion of astrocyte marker GFAP, neuronal marker Eno2 and pericyte marker PDGFRb by the brain endothelial cell isolation procedure was proven. Extension of the calculation of the depletion factor additionally including corresponding GAPDH Ct-values (ddCt) resulted in a 4-fold depletion of GFAP and Eno2 and a 2.5-fold depletion of PDGFRb. Data are presented as means ± SEM (n = 23), statistical significant difference to GAPDH was indicated by * ($p<0.05$, two-tailed Student's t-test).

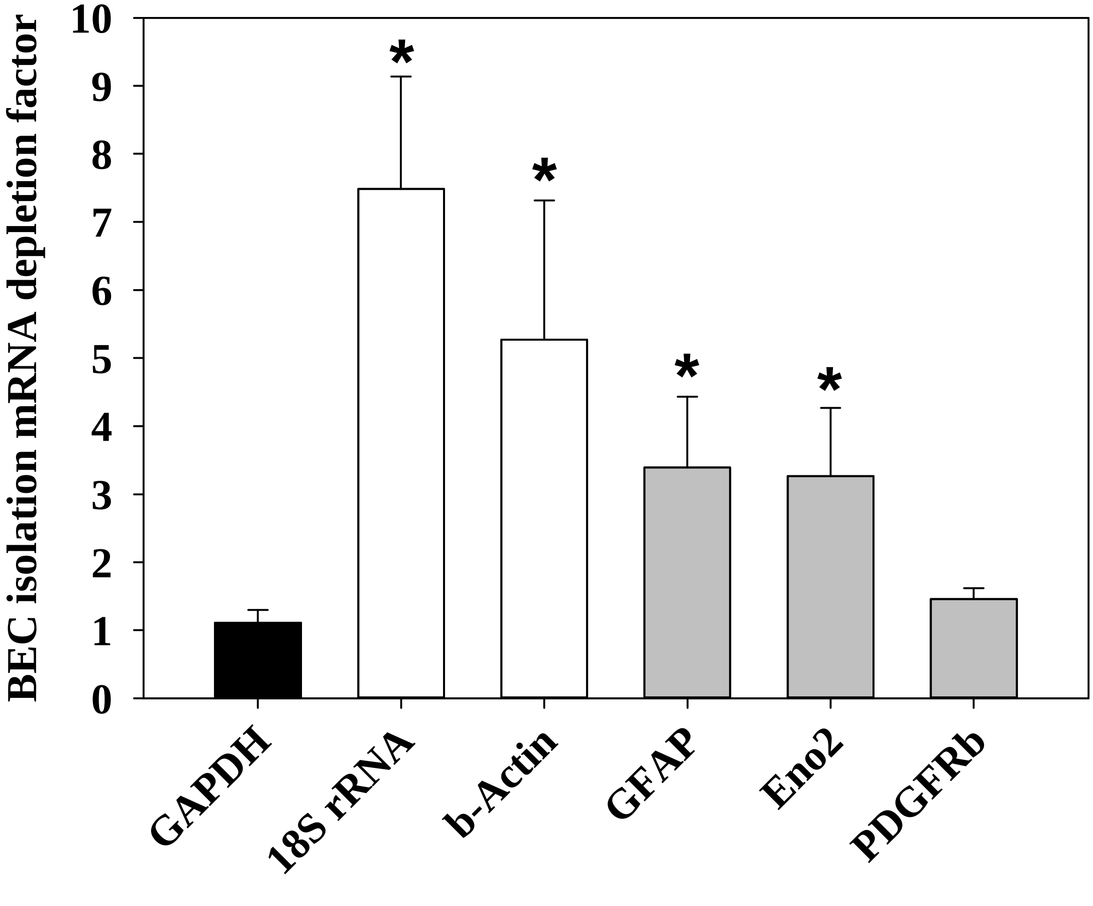

Supplement: S2 Fig — It was shown that mRNA of GAPDH was nearly unchanged in total brain versus brain endothelial fraction samples, whereas other targets often used as endogenous controls such as 18S rRNA and b-Actin were significantly depleted. Moreover, successful depletion of astrocyte marker GFAP, neuronal marker Eno2 and pericyte marker PDGFRb by the brain endothelial cell isolation procedure was proven. Extension of the calculation of the depletion factor additionally including corresponding GAPDH Ct-values (ddCt) resulted in a 4-fold depletion of GFAP and Eno2 and a 2.5-fold depletion of PDGFRb. Data are presented as means ± SEM (n = 23), statistical significant difference to GAPDH was indicated by * (p<0.05, two-tailed Student’s t-test). (PDF) [file pone.0136221.s003.pdf]
