# Supplementary material for: Multiple Antenatal Dexamethasone Treatment Alters Brain Vessel Differentiation in Newborn Mouse Pups
Source: PLoS One. 2015 Aug 14;10(8):e0136221. doi: 10.1371/journal.pone.0136221 (PMC4537167; doi:10.1371/journal.pone.0136221)

S3 Fig.: Effects of maternal DEX-treatment on receptor and transporter expression in total brains as well as brain endothelial fractions of PN10 pups. Data are presented as means ± SEM; for mRNA data: n = 5-6 biological samples, at PN10 one brain represented one biological sample; for western blotting: n = 6, at PN10 one brain represented one biological sample. Biological samples were collected from at least three different litters; $: p<0.05 (two-tailed Student's t-test). GR = glucocorticoid receptor; NR1 = NR1 subunit of N-methyl-D-aspartate receptor; Abcb1a = ABC-transporter b1a (=P-glycoprotein); Abcg2 = ABC-transporter g2 (=bcrp); Abcc4 = ABC-transporter c4 (=Mrp4); Glut1 = glucose transporter 1 (=Slc2a1), Mct1 = monocarboxylic acid transporter 1 (=Slc16a1).

Postnatal day 10

**Total brain – mRNA expression**				
target	1x NaCl	1x DEX	3x NaCl	3x DEX
**receptors**				
GR	1.00 ± 0.03	0.93 ± 0.04	1.00 ± 0.02	0.71 ± 0.24
NR1	1.00 ± 0.06	1.07 ± 0.09	1.00 ± 0.03	0.75 ± 0.28
**transporters**				
Abcb1a	1.00 ± 0.02	0.92 ± 0.11	1.00 ± 0.02	0.60 ± 0.11[$]
Abcg2	1.00 ± 0.01	0.92 ± 0.02[$]	1.00 ± 0.02	0.72 ± 0.23
Abcc4	1.00 ± 0.05	1.32 ± 0.22	1.00 ± 0.03	0.65 ± 0.19
Glut1	1.00 ± 0.09	0.99 ± 0.09	1.00 ± 0.03	0.67 ± 0.19
Mct1	1.00 ± 0.001	0.96 ± 0.06	1.00 ± 0.04	0.62 ± 0.19
**Total brain – protein expression**				
target	1x NaCl	1x DEX	3x NaCl	3x DEX
**receptors**				
GR	1.00 ± 0.14	0.90 ± 0.16	1.00 ± 0.07	1.00 ± 0.06
NR1	1.00 ± 0.09	0.84 ± 0.07	1.00 ± 0.06	0.69 ± 0.04[$]
**transporters**				
Abcg2	1.00 ± 0.07	0.95 ± 0.16	1.00 ± 0.25	0.80 ± 0.13
Glut1	1.00 ± 0.04	0.99 ± 0.09	1.00 ± 0.06	0.75 ± 0.03[$]
**Brain endothelial cells – mRNA expression**				
target	1x NaCl	1x DEX	3x NaCl	3x DEX
**receptors**				
GR	1.00 ± 0.11	0.94 ± 0.15	1.00 ± 0.04	0.65 ± 0.19
NR1	1.00 ± 0.01	0.86 ± 0.13	1.00 ± 0.05	0.61 ± 0.22
**transporters**				
Abcb1a	1.00 ± 0.14	0.88 ± 0.33	1.00 ± 0.07	0.72 ± 0.26
Abcg2	1.00 ± 0.11	0.71 ± 0.09	1.00 ± 0.03	0.73 ± 0.26
Abcc4	1.00 ± 0.15	1.05 ± 0.22	1.00 ± 0.08	0.49 ± 0.14[$]
Glut1	1.00 ± 0.18	0.68 ± 0.16	1.00 ± 0.04	0.77 ± 0.27
Mct1	1.00 ± 0.14	0.73 ± 0.16	1.00 ± 0.08	0.72 ± 0.25

western blots of total brain samples:

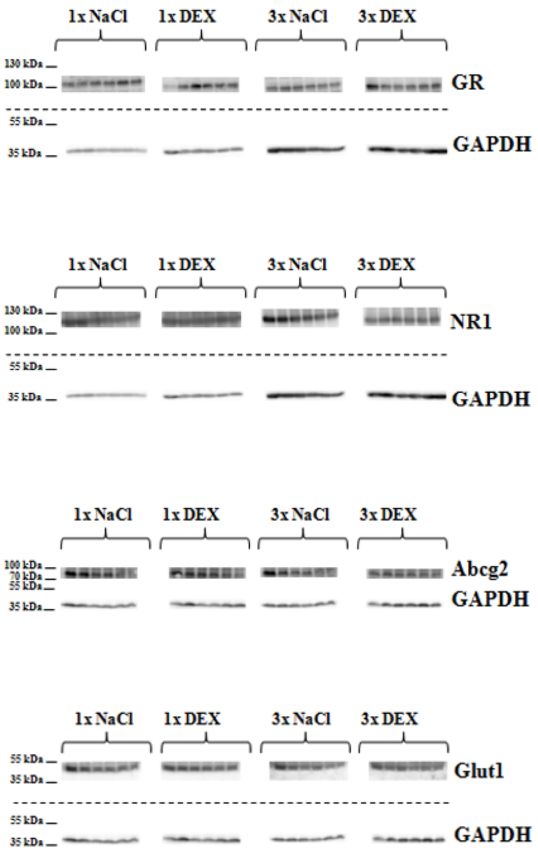

Supplement: S3 Fig — Data are presented as means ± SEM; for mRNA data: n = 5–6 biological samples, at PN10 one brain represented one biological sample; for western blotting: n = 6, at PN10 one brain represented one biological sample. Biological samples were collected from at least three different litters; $: p<0.05 (two-tailed Student’s t-test). GR = glucocorticoid receptor; NR1 = NR1 subunit of N-methyl-D-aspartate receptor; Abcb1a = ABC-transporter b1a (= P-glycoprotein); Abcg2 = ABC-transporter g2 (= bcrp); Abcc4 = ABC-transporter c4 (= Mrp4); Glut1 = glucose transporter 1 (= Slc2a1), Mct1 = monocarboxylic acid transporter 1 (= Slc16a1). (PDF) [file pone.0136221.s004.pdf]
