# Supplementary material for: Multiple Antenatal Dexamethasone Treatment Alters Brain Vessel Differentiation in Newborn Mouse Pups
Source: PLoS One. 2015 Aug 14;10(8):e0136221. doi: 10.1371/journal.pone.0136221 (PMC4537167; doi:10.1371/journal.pone.0136221)
Supplement: S1 Table — (PDF) [file pone.0136221.s005.pdf]

S1 Table: List of Taqman-probes used for qPCR

Target	Taqman® probe identification number
18SrRNA	EUK18SrRNA, 4352930-0810022
Abcb1a	Mm00440761_m1
Abcc4	Mm01226380_m1
Abcg2	Mm00496364_m1
Angiopoietin-1	Mm00456503_m1
Angiopoietin-2	Mm00545822_m1
Axin-2	Mm00443610_m1
β-actin	Mm01205647_g1
Claudin-3	Mm00515499_s1
Claudin-5	Mm00727012_s1
Eno2	Mm00469062_m1
GAPDH	Mm99999915_g1
GFAP	Mm01253033_m1
Glut-1 (Slc2a1)	Mm01192270_m1
GR (Nr3c1)	Mm00433832_m1
Grin1 (NR1)	Mm00433800_m1
Kdr (VEGFR2)	Mm00440099_m1
Mct1 (Slc16a1)	Mm00436566_m1
Neuropilin-1	Mm00435379_m1
Occludin	Mm00500912_m1
PDGFRb	Mm00435546_m1
Pecam-1	Mm01242584_m1
PTCH1	Mm00436026_m1
Shh	Mm00436528_m1
Sox-18	Mm00656049_gH
Tek (Tie-2)	Mm00443243_m1
Tjp1 (ZO-1)	Mm01320637_m1
Vegfa	Mm01281449_m1
