# Supplementary material for: Multiple Antenatal Dexamethasone Treatment Alters Brain Vessel Differentiation in Newborn Mouse Pups
Source: PLoS One. 2015 Aug 14;10(8):e0136221. doi: 10.1371/journal.pone.0136221 (PMC4537167; doi:10.1371/journal.pone.0136221)
Supplement: S2 Table — (PDF) [file pone.0136221.s006.pdf]

S2 Table: List of antibodies used for western blotting and immunofluorescence microscopy

Target	Product number, Company	Species	Application dilution
Abcg2	ab24115 (BXP53), Abcam	rat	1:100 for WB
Claudin-3	34-1700, Zymed®, Invitrogen	rabbit	1:100 for WB
Claudin-5	34-1600, Zymed®, Invitrogen	rabbit	1:200 for IF, 1:200 for WB
GADPH	MAB374, Millipore	mouse	1:2000 for WB
Glut-1	07-1401, Millipore	rabbit	1:100 for WB
GR	sc-8992, Santa Cruz	rabbit	1:200 for WB
Grin 1 (NR1)	sc-9058, Santa Cruz	rabbit	1:100 for WB
Occludin	71-5000, Zymed®, Invitrogen	Rabbit	1:400 for WB
Pecam-1	553370, BD Pharmingen	rat	1:100 for IF
ZO-1	40-2300, Zymed®, Invitrogen	rabbit	1:100 for WB
HRP-anti-mouse	LNA931V/AG, GE Healthcare UK ltd.		1:5000 for WB
HRP anti-rabbit	LNA934V/AG, GE Healthcare UK ltd		1:5000 for WB
HRP anti-rat	61-9520, Invitrogen		1:5000 for WB
anti-rat IgG Alexa Fluor 488	A11006, Invitrogen	goat	1:200 for IF
anti-rabbit IgG Alexa Fluor 568	A11011, Invitrogen	goat	1:200 for IF

WB = western blotting, IF = immunofluorescence microscopy
