# Supplementary material for: Multiple Antenatal Dexamethasone Treatment Alters Brain Vessel Differentiation in Newborn Mouse Pups
Source: PLoS One. 2015 Aug 14;10(8):e0136221. doi: 10.1371/journal.pone.0136221 (PMC4537167; doi:10.1371/journal.pone.0136221)
Supplement: S3 Table — (PDF) [file pone.0136221.s007.pdf]

S3 Table: Effects of antenatal DEX-treatment on cell marker expression of Pecam-1/CD31 (brain endothelial cells), PDGFRb (pericytes), GFAP (astrocytes), Eno2 (neurons) in total brains as well as brain endothelial fractions of PN4 and PN10 pups.

Postnatal day 4

Total brain – mRNA expression				
target	1x NaCl	1x DEX	3x NaCl	3x DEX
Pecam-1	1.00 ± 0.16	1.14 ± 0.34	1 ± 0	0.48 ± 0.13$
PDGFRb	1.00 ± 0.14	1.10 ± 0.28	1 ± 0	0.59 ± 0.22
GFAP	1.00 ± 0.004	1.09 ± 0.14	1 ± 0	0.60 ± 0.19
Eno2	1.00 ± 0.01	1.29 ± 0.26	1 ± 0	0.55 ± 0.16$
**Brain endothelial cells – mRNA expression**				
target	1x NaCl	1x DEX	3x NaCl	3x DEX
Pecam-1	1.00 ± 0.15	1.99 ± 0.56	1 ± 0	0.50 ± 0.13$
PDGFRb	1.00 ± 0.14	1.77 ± 0.28	1 ± 0	0.50 ± 0.13$
GFAP	1.00 ± 0.02	2.25 ± 0.21$	1 ± 0	0.57 ± 0.14
Eno2	1.00 ± 0.005	2.88 ± 0.38$	1 ± 0	0.50 ± 0.10$

Postnatal day 10

Total brain – mRNA expression				
target	1x NaCl	1x DEX	3x NaCl	3x DEX
Pecam-1	1.00 ± 0.02	1.27 ± 0.27	1.00 ± 0.06	0.68 ± 0.17
PDGFRb	1.00 ± 0.01	1.04 ± 0.07	1.00 ± 0.04	0.65 ± 0.18
GFAP	1.00 ± 0.07	0.85 ± 0.06	1.00 ± 0.004	0.62 ± 0.19
Eno2	1.00 ± 0.05	1.18 ± 0.09	1.00 ± 0.02	0.72 ± 0.24
**Brain endothelial cells – mRNA expression**				
target	1x NaCl	1x DEX	3x NaCl	3x DEX
Pecam-1	1.00 ± 0.21	0.75 ± 0.20	1.00 ± 0.06	0.86 ± 0.29
PDGFRb	1.00 ± 0.08	0.72 ± 0.06*	1.00 ± 0.06	0.68 ± 0.24
GFAP	1.00 ± 0.05	0.88 ± 0.12	1.00 ± 0.04	0.75 ± 0.26
Eno2	1.00 ± 0.18	0.93 ± 0.18	1.00 ± 0.02	0.69 ± 0.26

Data are presented as mean ± SEM; n=5-6 biological samples, at PN4 2-3 brains from one litter were pooled to one biological sample, biological samples were collected from at least three different litters; $:p<0.05, *: p=0.06.
